# Analectic

**Published:** 1828-06

**Authors:** 


### MISCELLANEOUS INTELLIGENCE.

### 1. ANALECTIC

1. *Peculiar Disease of the Heart.*—Our readers will remember that, within the last year, we described three or four cases of a peculiar disease of the heart—one in the person of the late celebrated Talma—another, from our own practice, the case of the late General Kyd; and one by Cruveilhier. The indefatigable Breschet has dedicated a long article to this subject in the last number of the REPERTOIRE, in which he has collected nearly a dozen of instances of this very curious and rare disease. We shall endeavor to seize the more interesting particulars of this paper for the information of our readers.

1. The first case which M. Breschet has been able to find on record, is one related by Walter, the father, in the year 1759. The patient was a merchant, 50 years of age, who had, for many years before his death, complained of præcordial anxiety and palpitation of the heart. A pouch was found arising from the left ventricle.

2. After alluding to Dr. Baillie's case, the following is detailed from Zannini, published in 1816. A gondolier at the age of 19 years, fell and bruised his chest. At the age of 25, he became affected with a complaint of the chest, attended with pain in the side, difficult breathing, cough, and bad expectoration, which symptoms were relieved by bleeding. Soon after this, he felt, for the first time, a pulsation in the *right* side of his chest, attended with a sensation as if a body was moving up and down there. He continued his avocations, and drank much wine. Two years afterwards, he became annoyed with a pain in the region of the heart, which was relieved by bleeding, but continued to return from time to time, especially on using much exertion. He had a disagreeable pulsation at the pit of the stomach. He lived two years in this condition, still pursuing his avocations, when his suffering were a little mitigated. Having attained the age of 29 years, he suddenly expired one day while making some corporeal exertion, after eating rather heartily.

On dissection, the lungs were found healthy. The pericardium contained a few ounces of yellow serum. From the left ventricle of the heart there went off a pouch, the size of a man's fist, which was adherent to the pericardium. This tumour opened into the cavity of the ventricle, and contained coagulated blood. The parietes of the aneurismal production varied in thickness, from three quarters of an inch to an inch and a half. There was nothing else particular in the dissection.

3. This was a negro, who was received into La Charite, on the 17th October, 1796, in the agonies of death. He died the next day, after a most profuse nasal hæmorrhage. On dissection, it was observed that the heart was of its natural size; but, from the left ventricle, there went off a tumour, nearly as large as the heart itself. The parietes of this aneurismal tumour were of a cartilaginous consistence, although they preserved the appearance of muscle. The interior of the tumour presented several albuminous layers, of considerable density, exactly resembling those seen in an arterial aneurism, except that they were more pale. The cavity of the tumour communicated, by a rather small aperture, with that of the ventricle. The aneurism appeared evidently to be formed between the muscular substance of the heart and its pericardinal covering. The mitral valve was thickened and ossified. This case was recorded in the second edition of Corvisart's work. Laennec never saw an instance of the disease, and only touches on the subject, placing the disease under the head of "Dilatations partielles du Cœur."

4. Two cases of this malady were observed by M. Berard, in the dissecting room of La Charite, and whose histories were therefore, unknown. The second of these cases offered some remarkable phenomena. The man appeared to have been about 55 years of age, and the body was very fat. On opening the chest, the pericardium presented a most enormous size. The heart was prodigiously dilated, and, at the same time thickened in its parietes. From the summit of the left ventricle there went off an aneurismal pouch, whose interior was covered with concentric layers of organized coagulable lymph, but not of very long standing apparently.

5. The case which we gave in No. 14 of this Series, page 401 as related by M. Cruveilhier, is next introduced by M. Breschet, and this we shall, of course, pass over.

6. The next instance came under M. Breschet's own observation. On the 27th of March, a soldier, aged 49 years, was received into the clinical ward, stating that he had suffered, for six or eight months, from oppression and want of breath, especially on using exercise. The limbs and belly were now infiltrated—he was unable to lie down in bed—the left ventricle of the heart communicated a considerable impulsion to the cylinder, and each contraction was attended with a whizzing noise, (bruit de soufflet) which was very distinct, but which, however, diminished afterwards, and finally disappeared. The pulse was small, and sometimes unequal. Diuretics, purgatives, &c. were employed with the view of removing the consecutive dropsical swellings. On the night of the 18th May, the patient suddenly became insensible, and complete hemiplegia of the right side ensued.—Bleeding, leeching, &c. were used, but the hemiplegia continued, although some degree of the intellectual functions was restored. He died on the 23d May.

*Dissection.* There was serous effusion between the membranes, and in the ventricles of the brain. The right corpus striatum was of a livid colour, and completely softened almost into a fluid. This softening was exactly confined to the corpus striatum, and no other morbid appearance was seen in any part of the brain or cerebellum. The heart was nearly double its natural size, and, from the left ventricle, a small pouch went off, such as has been described. The great size of the heart was occasioned by the dilatation of the left ventricle, whose parietes, however, could hardly be said to be thickened. The tricuspid valve was in a state of induration approaching ossification. The interior of the pouch presented concentric layers of dense fibrine. The liver was granulated.

7. The case of Talma forms the next instance adduced by M. Breschet, but this has been already noticed in our own Journal.

### GENERAL OBSERVATIONS AND CONCLUSIONS.

Our author remarks that the word *aneurism* has given rise to numerous altercations, discussions, and erroneous deductions. By this term have been understood various and essentially different diseases of the heart and of the arteries. It is also remarkable, that we have admitted the existence of certain morbid conditions of the heart which we have denied to the arteries and *vice versa.* Thus, every one will allow that the chambers of the heart may become dilated, with or without *thickening*—with or without *attenuation* of their parietes; but it has been denied that there can be an aneurism of the heart produced by rupture of the fleshy parietes, and dilatation of its membranous envelopes. In respect to the arteries, it has been contended that there can be no such thing as true aneurism—that is, a general dilatation of the whole cylinder of the vessel; but that, in all cases, there is a morbid condition of the inner and middle coats, with laceration of these tissues, and the formation of a tumour on one side of the vessel, in which is contained blood, or layer after layer of fibrine. Sennertus, Fab. Hildanus, and Scarpa, maintained this doctrine. But it is too exclusive. There can be no doubt that a diseased condition of the arterial coats, with partial laceration or dilation of them into a pouch, is the more usual form of aneurism; but to deny that there can be a general dilatation of the calibre of the vessel, without laceration or disease of the coats, is an error. In 14 examinations of aneurismal tumours, Burns found but one in which the doctrine of Scarpa was impugned. Still this one exception in 14 cases proves that the rule is not exclusive. The present paper, and others which we have laid before our readers, evince that the heart is liable to the same kind of disease as the arteries—namely, partial attenuation, rupture, or dilatation of the parietes of some of its chambers, with the consequent formation of a pouch, containing coagulated blood, or layers of fibrine. Observation has proved, that these aneurismal pouches are almost always found to go off from the left ventricle of the heart. What can be the cause of this? M. Portal is of opinion that these pouches may form without any previous disease in the parietes of the chamber. He thinks the disease is more the consequence of violent contraction, than of dilatation of the chamber. There can be no doubt that these pouches are often seen where the rest of the parietes are in a state of hypertrophy; but M. Breschet is of opinion, and we agree with him, that, in all probability, there is an inequality of power in the different parts of the chamber, previously to the formation of these pouches. It is very curious, however, that aneurism by dilatation should be extremely common in the heart, and comparatively rare in the arteries; while, on the other hand, the aneurism by rupture or partial dilatation of the parietes is the usual form of the disease in the arteries, and comparatively rare in the heart. The reason for this difference is not very apparent. M. Breschet has attempted to lay down some symptoms as tending to show the existence of this disease; but we consider them as quite uncertain—if not absolutely erroneous. It rarely falls to the lot of a medical man to see a specimen of this curious disease; but the one which we published—that of General Kyd, was not accompanied by any of those symptoms which M. Breschet mentions—indeed the patient never exhibited any symptoms during life, which indicated organic affection of the heart at all. Still we hold it extremely desirable that we should be acquainted with all the forms of organic disease that may be presented to our view in the course of pathalogical investigations; and with this view we have presented our readers with a succinct account of our learned author's paper on the above subject. The final conclusions to which M. Breschet comes are the following:

1mo. The heart is liable to be affected by a disease hitherto undescribed by nosographers or practitioners—2dly. That the disease is chiefly found affecting the left ventricle—3dly. That the *summit* of this ventricle is the principal seat of the disease—4thly. That the tumour is aneurismal. 5thly. That this aneurism, from the mode of its formation, the state of its parietes, and the parts contained in the cyst, may be considered analogous to the "false consecutive aneurism of arteries," in the sense employed by Scarpa—6thly. That the heart is liable to the same kind of aneurism which we commonly meet with in the arteries—a circumstance that might be reasonably expected, from their structure and functions.—REPERTOIR.—*Med. Ch. Rev. for Jan.* 1828.—p. 151.

2. *Intestinal Invaginations.*—Intestinal invaginations or intussusceptions are often seen in young subjects who have died of other diseases, and appear to take place in the act of dying, from some convulsive or inordinate movements of the muscular fibres of the intestines. They are thus, probably, of no consequence whatever. But when a portion of small testine is inverted into the colon—or when a portion of this last is inverted on itself, it becomes a very serious affair, and most generally fatal. It is with the view of furnishing some data for distinguishing this last and dangerous species of invagination, that M. Buet brings forward the two following cases, from the wards of Dr. Husson, in the HOTEL DIEU of Paris.

*Case* 1. On the 17th of April, 1825, there was received into the above-mentioned hospital, a man, aged 40 years, formerly a soldier, but now a tradesman. This man, of large stature, and strong constitution, presented the following symptoms when received into hospital: Abdomen very hard and distended, as well as painful on pressure, especially in the *left* iliac fossa, the *right* being rather depressed, and void of pain when pressed. The abdominal muscles appeared in strong action; pains in the loins: frequent eructations; vomiting of yellowish matters; constant inclination to stool; colicky and dragging pains in the belly; constriction about the throat. During the paroxysms of colicky pains, the left iliac region presented the figure of a large convolution of intestine, bent on itself. The poor man's countenance was indicative of great suffering; the tongue was furred; much thirst; slow pulse. The patient stated the following particulars of his illness. During ten years' sojourn at Rochefort, he had experienced several attacks of intermittent fever, which had been treated by vomits, purgatives, and tonics, &c. Afterwards, he was very subject to vomiting and diarrhœa. On the 20th December, 1824, after some muscular exertion, he felt, in the *right* iliac fossa, a very acute pain, followed by colick, vomiting, and purging. These attacks came on frequently afterwards, with intervals of ease. He had occasionally discharges of blood from the bowels. The means that had been employed were leeches, fomentations, demulcent drinks, and the like. M. Husson considered the case as hopeless, and that nothing could be done but soothe the remainder of the patient's days. Thirty leeches were applied to the anus, anodyne lavements were thrown up, and fomentations were ordered to the abdomen. Little or no relief was obtained by these means. On the 23d of April, while straining at stool, the patient felt as if something had given way in his abdomen, and, in a few minutes more, he became very ill, the abdomen becoming inflated, and exquisitely tender to the touch. He died at eight o'clock the same evening.

*Dissection.* The peritoneal lining of the abdomen was every where inflamed, but there was no extravasation. Patches of recent coagulable lymph were, however, numerous. The small intestines were excessively distended with gas. Neither the cæcum nor ascending portion of colon could be found; they were carried forward, and inclosed in the last half of the transverse arch, and in the sigmoid flexure of the colon, which was as thick as a man's arm, and hard or solid to the touch. On examination, the cæcum was found lodged in the sigmoid flexure, in the iliac fossa. The invaginated portion consisted, therefore, of three parietes, as may be easily conceived, from the mode of introversion, since a portion of ileum was necessarily carried in with the cæcum and ascending arch of the colon. The invaginated parts were agglutinated together, and, in some places, gangrene had commenced. There was nothing particular in the other portions of intestine, beyond the distention, which might be naturally expected above so serious an obstruction to the evacuation of the bowels.

This case, M. Buet thinks, illustrates the effects of long-continued and drastic purgatives, to the intemperate use of which he attributes the invagination above-described. This case also shows, that an intus susception of great extent may exist for a long time, and yet a passage for liquid fæces may still obtain. Up to the day before the patient's death, he had fluid evacuations from his bowels.

*Case* 2. Pradier, aged 22, who had been in the hospital at Clermont, for vomiting and diarrhœa, with colicky pains, came to Paris, in July, 1825, in a very bad stale of health, and entered the Hotel Dieu, on the 8th of August, under Dr. Husson. The patient's symptoms, at this time, did not portend the existence of so serious a malady as that which actually obtained. He had colicky pains and diarrhœa, with intervals of perfect ease, and his complexion was nearly natural. He was ordered emollient fomentations, diluents, and lavements. On the 12th and 13th of the same month, the symptoms were observed to be aggravated; the features were sharp; the pulse concentrated; and the iliac fossa of the left side was very painful on pressure. Leeches and opiates were prescribed. 14th. The pain was insupportable, and the patient felt as if something was tearing in his inside He had nausea and vomitings, with spasmodic contractions of the fingers and other parts. 16th. Symptoms of peritoneal inflammation were evident, and leeches in great numbers were applied; but the disease went on unchecked, and death terminated his sufferings on the 18th of the same month.

*Dissection.*—The peritoneum was highly injected, and there were numerous adhesions and false membranes seen in different parts, but no effusion of extravasation. Neither cæcum, ascending or transverse arch of the colon, could be found. The descending portion was as thick as a man's arm, and very hard. On performing moderate traction, it was easily discovered that the cæcum and ascending and transverse arches of the colon, together with a portion of ileum, were engulphed in the descending portion of the colon. The cæcum was found in a state of gangrene.—*Archives Generalis.*—*Ibid.* p. 156.

3. *Endermic Medication.*—In spite of all the controversies which have taken place respecting the existence of a capability in the vessels of the skin to absorb substances applied to them—the general conviction, supported now by incontestable facts, is, that such absorbant power does actually exist—especially when the epidermis is removed. The method of introducing medicinal agents through this channel is by no means a mere matter of physiological curiosity. It may be turned to great advantage in many cases, where there are strong objections to the administration of medicines by the mouth. Thus, a person may become the subject of ague while labouring under chronic inflammation of the stomach or bowels—and, consequently, where bark or arsenic would be injurious. In such a case, the sulphat of quinine may be applied to the denuded surface of a blister, and the ague will be stopped without any irritation being given to the stomach or bowels. Again, in cases of children, who have a natural repugnance to medicines, and especially to bitter medicines, the endermic medication offers a very convenient resource.

In a late Number of the Revue Medicale, the younger Martin has published several cases of intermittent fever, treated, at the Hotel Dieu, by sulphat of quinine, applied with cerate to blistered surfaces. At first, he applied the powder over the denudeu cutis, but it occasioned too much irritation, and the paroxysms were not stopped. He then mixed the quinine with cerate, and the object was obtained, without any local inflammation or pain being produced. A single case in illustration will be sufficient for our purpose.

*Case.* Leon, a soldier, of the 1st regiment of Light Infantry had been in the hospital upwards of four months, when M. Martin took his turn of duty, on the 1st of December, 1826. He had had a pernicious remittent fever, attended with several relapses, by which he was reduced to a state of great debility and emaciation. He was also affected with dry cough, and was compelled to keep his bed, from the general state of marasmus to which he was brought by his disease. By proper means, the cough was mitigated, and the patient began to recruit a little, when, on the morning of the 20th December, he was seized with a regular fit of ague, which left the cough in a state of exasperation. The next day, being apyrectic, a blister was applied to his arm. On the third day, in the morning, the blister was removed, and the surface dressed with cerate, in which was incorporated six grains of the sulphat of quinine. That day the paroxysm was much milder, and at somewhat a later hour. The quinine was continued, six grains daily in the cerate, and fever returned no more.

We need not multiply cases, as the foregoing presents an example that will be easily imitated, where it is deemed necessary. We apprehend that this plan may be applicable to a great many morbid conditions of the system, where the internal administration of remedies would be inconvenient.—*Ibid.* p. 158.

